# Effectiveness of exercise interventions in animal models of multiple sclerosis

**DOI:** 10.3389/fmed.2023.1143766

**Published:** 2023-03-30

**Authors:** Abdolhossein Parnow, Muthanna Hafedh, Ikuo Tsunoda, Darpan I. Patel, Julien S. Baker, Ayoub Saeidi, Sovan Bagchi, Pallav Sengupta, Sulagna Dutta, Edyta Łuszczki, Artur Stolarczyk, Łukasz Oleksy, Maisa Hamed Al Kiyumi, Ismail Laher, Hassane Zouhal

**Affiliations:** ^1^Department of Sport Biological Sciences, Physical Education and Sports Sciences Faculty, Razi University, Kermanshah, Iran; ^2^Department of Exercise Physiology, General Directorate of Education Basrah, Basrah, Iraq; ^3^Department of Sports Activities, College of Adm&Eco/Qurna, University of Basrah, Basrah, Iraq; ^4^Department of Microbiology, Faculty of Medicine, Kindai University, Osaka, Japan; ^5^School of Nursing, University of Texas Health Science Center at San Antonio, San Antonio, TX, United States; ^6^Department of Sport, Physical Education and Health, Centre for Health and Exercise Science Research, Hong Kong Baptist University, Kowloon, Hong Kong SAR, China; ^7^Department of Physical Education and Sport Sciences, Faculty of Humanities and Social Sciences, University of Kurdistan, Sanandaj, Iran; ^8^Department of Biomedical Sciences, Gulf Medical University, Ajman, United Arab Emirates; ^9^School of Medical Sciences, Bharath Institute of Higher Education and Research (BIHER), Chennai, India; ^10^Institute of Health Sciences, Medical College of Rzeszów University, Rzeszów, Poland; ^11^Department of Orthopedics and Rehabilitation, Medical University of Warsaw, Warsaw, Poland; ^12^Department of Physiotherapy, Faculty of Health Sciences, Jagiellonian University Medical College, Kraków, Poland; ^13^Department of Family Medicine and Public Health, Sultan Qaboos University Hospital, Sultan Qaboos University, Muscat, Oman; ^14^Department of Anesthesiology, Pharmacology and Therapeutics, The University of British Columbia, Vancouver, BC, Canada; ^15^University of Rennes, M2S (Laboratoire Mouvement, Sport, Santé) - EA 1274, Rennes, France; ^16^Institute International des Sciences du Sport (2I2S), Irodouër, France

**Keywords:** EAE model, exercise training, multiple sclerosis, motor function, neurotrophin

## Abstract

Multiple sclerosis (MS) is associated with an impaired immune system that severely affects the spinal cord and brain, and which is marked by progressive inflammatory demyelination. Patients with MS may benefit from exercise training as a suggested course of treatment. The most commonly used animal models of studies on MS are experimental autoimmune/allergic encephalomyelitis (EAE) models. The present review intends to concisely discuss the interventions using EAE models to understand the effectiveness of exercise as treatment for MS patients and thereby provide clear perspective for future research and MS management. For the present literature review, relevant published articles on EAE animal models that reported the impacts of exercise on MS, were extracted from various databases. Existing literature support the concept that an exercise regimen can reduce the severity of some of the clinical manifestations of EAE, including neurological signs, motor function, pain, and cognitive deficits. Further results demonstrate the mechanisms of EAE suppression with information relating to the immune system, demyelination, regeneration, and exercise in EAE. The role for neurotrophic factors has also been investigated. Analyzing the existing reports, this literature review infers that EAE is a suitable animal model that can help researchers develop further understanding and treatments for MS. Besides, findings from previous animal studies supports the contention that exercise assists in ameliorating MS progression.

## Introduction

Multiple sclerosis (MS) is an inflammatory demyelinating illness that impairs the central nervous system (CNS). MS presents a complex etiology not only impairing the immune mechanisms but also disrupting the neurons as well as the axons and oligodendrocytes (1). Although extensive research has determined that MS is mainly associated with immune-deregulations, the complete etiopathology of MS is yet to be explored. It is suggested that major factors in the disease susceptibility are the combination of several environmental variables and numerous gene interactions. Around the world, approximately 2.8 million people suffer from MS, and women are twice as likely to have the condition as men (2). The disease has been classified into three categories: primary progressive MS (PP-MS), relapsing remitting MS (RR-MS) and secondary progressive MS (SP-MS) (2, 3). Most MS patients initially belong to the RR category initially affects, then progresses to SP-MS with accumulated neurologic defects (4). The PP type affects 10–15% of patients and has been defined as a progressive disease with no remission (5).

Some symptoms/signs of MS act as obstacles against the routine activities of daily living. These factors include ambulatory difficulty, disequilibrium (balance impairment), heat intolerance, muscle weakness, spasticity, cognitive impairment, and fatigue (6). In contrary, there are reports suggesting influence of psychosocial factors affecting MS patients such as poor education, celibate life, smoking (7), depression, or anxiety (8).

Generally, an effective exercise prescription can lead to significant improvements in various aspects of health, including cardiorespiratory fitness, muscle strength, flexibility (9), cognitive function (10), and quality of life (11). Additionally, it is generally known that engaging in regular physical exercise benefits the neurological system in a variety of ways, including neuroprotection, increased plasticity of the neurons and enhanced learning capacity (12). In patients with MS, exercise training could be considered a complementary therapy for classical treatment (13). For many years, however, fresh diagnosed MS patients are suggested by their Physicians to avoid any exercise and physical activity because of the fear of exacerbations. Recently, exercise and physical training have been proposed as a possible solution to delay the progression of MS (14–18). This process can be achieved by reaching and maintaining an optimal patient activity level. The increase in physical function provides physiological and mental health benefits without any concerns relating to exacerbating the disease symptoms or relapse in MS (19, 20).

Previously, Klaren et al. (21) reviewed how exercise training impacted animal models outlining the physiological and therapeutic aspects, providing new insights into methodological approaches and outcomes related to exercise training that utilized animal models of MS. In this review, we aimed to expand on the previous work and present a summary of animal model studies that reported the impacts of exercise on MS.

## MS research with animal models

Numerous animal models have been established in recent years ti mimic the clinical symptoms and neuropathology of MS. Using animal models allows investigators to manipulate the disease course and study the effects on neuropathology using CNS samples that may not be feasible in human clinical trials. Currently, a wide spectrum of animal models for MS with different specific features is available. Overall, the animals models can be classified into three different types, which can be used for different aspects of research, they include: (1) experimental autoimmune (or allergic) encephalomyelitis (EAE), (2) virus-infected models, such as murine hepatitis virus (MHV) and Theiler’s murine encephalomyelitis virus (TMEV) models, and (3) toxin-induced models of demyelination, that include the cuprizone model and focal demyelination induced by lysophosphatidylcholine (22).

The EAE animal model is the one that is utilized in MS research the majority of the time. EAE is the model that most accurately mimics the autoimmune etiology of MS. It is also an incredibly helpful tool for researching potential new treatments. It has directly contributed toward the development of several first-line treatments that target the inflammatory phase of the disease (23). As explained in the following paragraphs, it is convenient to induce EAE in animals. It is impractical to expect a single animal model to accurately represent the pathophysiology of MS given the genetic diversity of the patient population, and the variety of environmental factors that may affect the onset and development of the illness. Instead, each EAE model imitates a unique component of MS, and the variety of the EAE models is the key advantage of using these (24). Importantly, the EAE models have demonstrated to be very helpful for identifying and evaluating the effectiveness of the disease-modifying therapies. Particularly spontaneous EAE models show significant potential as a method for understanding the pathophysiology of MS and will be very helpful for testing drugs that might treat or prevent the illness (24).

Two ways of sensitization have been frequently employed to induce EAE in animals: active induction *via* myelin-antigen-sensitization and passive induction done *via* adoptive-transfer of myelin specific T lymphocytes in animals (25). “Active” Sensitization of sensitive animal strains with CNS homogenate or myelin protein/peptides, for example, myelin oligodendrocyte glycoprotein (MOG), myelin basic protein (MBP), and myelin proteolipid protein (PLP), induces EAE (26). Additionally, recipient mice can be used to develop “passive” or “adoptive”-transfer EAE (AT-EAE) when pathogenic myelin-specific T cells produced in active immunized donor animals, are transfer to it (26). A major difference between MS and EAE is that the latter requires an artificial sensitization to autoantigens using adjuvants such as complete Freund’s adjuvant and pertussis toxin. Although “spontaneous” EAE does not require such artificial sensitization, it employs transgenic mice having genes of T-cell receptors specific to myelin antigen (27).

## Exercise training and EAE model overview

Strength training or running on forced-treadmill (FTR) or voluntary wheel (VWR) are two exercise-based regimens that can be used with animal models. VWR in mice has been described by Manzanares et al. as intermittent, which is comparable to interval training in humans (28, 29). They also indicated several advantages for VWR, including: (a) Running is analogous to the natural running behavior of mice; (b) running is conducted in non-stressed conditions, in accordance with the animal’s natural rhythm, and does not involve direct investigator’s intervention, making it suitable for long-term studies (28). In addition, Huang et al. have tested various types of VWR, including intermittent VWR and continuous VWR (30). In other exercise regimen in mice models, including FTR and swimming, employ unpleasant stimuli to motivate active activity. Although it offers the benefit of regulating activity at repeatable rates and distances, it might not be entirely consistent with typical mouse behavior (29). Although VWR and FTR exercise regimens have been reported to be beneficial as a pre-conditioning tool for the CNS (31), FTR exercise has been demonstrated to be more effective in promoting neuroprotection (32, 33) by influencing brain metabolism.

**FIGURE 1:**
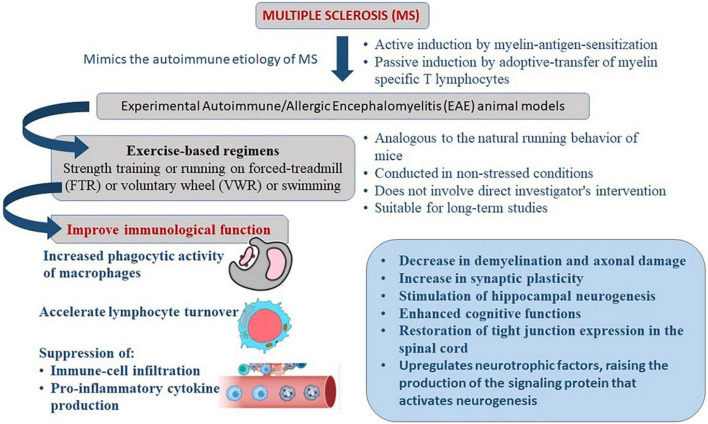
Mechanism of exercise mediated reduction in severity of clinical manifestations of experimental autoimmune/allergic encephalomyelitis (EAE) that mimics the immune-pathogenesis of multiple sclerosis (MS).

More than 3 weeks of exercise is necessary to promote the effects of stress response adaptation and neuroprotection (34–36). On mice, programs based on long-term exercise have been found to improve immunological function. The increased phagocytic activity of macrophages, for example, may be responsible for these consequences (37). Long-term physical activity appears to accelerate lymphocyte turnover through enhanced cell proliferation (38, 39). In EAE models, several studies have been conducted to explore the benefits of exercise based on immune-mediated mechanisms, including suppression of immune-cell infiltration and pro-inflammatory cytokine production as well as non-immune mechanisms. These include a decrease in demyelination and axonal damage, an increase in synaptic plasticity, the upregulation of neurotrophins, the stimulation of hippocampal neurogenesis, antioxidant effects, and the restoration of tight junction expression in the spinal cord (40–47) (Figure 1).

## Experimental design of exercise training on MS animal models

### Effect of exercise on the disease onset and clinical scores of EAE

Physical activity or exercise training has been reported to directly affect disease onset and clinical scores in EAE models. The search strategy resulted in 11 studies evaluating the effects of exercise training on outcomes of clinical amelioration and onset delays in EAE models. Nine studies have reported clinical improvements and onset delays related to the EAE model utilizing different modalities of exercise [i.e., FTR (47–49), VWR (40, 45, 46, 50), and swimming exercise (41, 51) and the duration of the physical activity period itself. However, in two studies (52, 53)], FTR and VWR had no significant impact on clinical symptoms, such as the frequency and severity of EAE relapses. In these studies, the amount of wheel- and treadmill-running exercise seemed to elicit no significant differences in clinical disability compared to sedentary conditions. For example, Patel and White reported no significant differences in clinical disability, brain mass and BDNF (53). These contradictions are perhaps due to the volume and/or intensity of activity training protocols. Moreover, these two studies’ insufficient amount of activity in the treadmill and wheel running conditions may be associated with different rodents strain, fatigue (resulting from muscle weakness in EAE) (54), depressive-like behaviors (a common symptom in EAE) (55).

### Effects of exercise on pain and cognitive deficits in EAE

More than 50% of MS patients experience sensory impairment and cognitive dysfunction, including learning and memory disturbances (56). We identified two reports analyzing the impacts of exercise on sensory and cognitive impairments in EAE (Table 1). In the first study, Benson et al. (40) indicated that EAE mice with 1 h/day of running on a voluntary wheel had improved pain hypersensitivity. In this study, C57BL/6 mice (*n* = 60) performed VWR exercise a week prior to the EAE induction and up to a day after induction. The authors observed that the VWR activity delayed the disease onset and decreased pain hypersensitivity. This phenomenon was corresponded with decreased inflammatory reactions in the spinal cord. In addition, the VWR resulted in decreased oxidative stress production in the spinal cord (40). In the second study, Kim et al. investigated the effects of exercise training on memory in C57BL/6 mice with EAE (44). The exercise intervention commenced using the following methods; exercise was initiated 20 days post-sensitization, consisted of 5 days a week, and 30 min daily for 4 weeks. Each week, the intensity of the workout (i.e., the speed of the treadmill) was raised by a little amount (2 m per minute in the first week; 3 m per minute in the second week; in the third week, it was 4 m per minute; followed by 5 m per minute in the fourth week). Mice were grouped into control/sham, EAE, and EAE + exercise (EAE + EX) groups. The investigation of cognition was conducted *via* a step-down avoidance apparatus. The results revealed that regular exercise alleviated memory deficits in EAE mice. The activation of the hippocampus region increases neurogenesis and inhibits apoptosis. Consequently, activation of the hippocampus increases cognitive performance, and exercise influences physiological and biochemical changes in the brain, notably in the region of the hippocampus. In addition, regular exercise enhanced neurogenesis marker proliferation in EAE animals and decreased apoptosis in their dentate gyrus (44). The study used BrdU as a biomarker for neurogenesis.

**TABLE 1:** Sample characteristics for studies of pain and cognitive decline.

References	Animal species	Cognitive apparatus	Type and duration of exercise intervention	Exercise starts prior of EAE induction	Exercise start post of EAE induction	Analysis	Result
Benson et al. (40)	C57BL/6 female mice, 12 weeks old	Von Frey hair	Voluntary wheel running, 8 days	1-week prior induction.	1 day post-induction	Pain	1 h/day of voluntary wheel running improved pain hypersensitivity.
Kim and Sung (97)	C57BL/6 female mice, 10-weeks old	Step-down avoidance	Treadmill exercise, 4 weeks		20 days post-induction	Memory	4 weeks of regular treadmill exercise Influence on improvement of memory in mice with EAE.

### Effect of exercise on motor function in EAE

It is noted that the mechanism of exercise involved in molecular and cellular responses in humans is difficult to establish since its manipulation and measuring process is not possible in humans in the same way that has been done in animals *via* the use of brain tissue samples (57, 58).

Recent advancements in neuroimaging have made it possible to do *in vivo* study in non-invasive manner on the impact of physical activity on structure and functions of larger circuits and regions (57, 58). A review article, for example, investigated the impact of cognitive and motor rehabilitation on neuroimaging outcomes in human and animal MS models (59). It used the term “morphology” in a broad sense to describe changes to the structures of the brain, which are frequently assessed by determining the integrity of the white matter or the volume of white and/or gray matter. In addition, separate studies demonstrated that brain morphology, functional and/or structural brain changes (60) relate to cognitive function to physical activity or fitness influences (57, 58) and balance and posture issues have been observed in patients with MS and EAE model (61–63).

Three studies quantified the effects of exercise training on motor function in EAE. One study was excluded because the application of wheel running was used as a scale for fatigue assessment and not for exercise adaptation (64) (Table 2). Two studies measured motor function following exercise training using a Catwalk (62) or motor Rotarod (63) apparatus to investigate the motor impairment and gait parameters in C57BL/6 mice. Both studies used the same exercise protocol with few variables: 5 weeks (5 days/week) of FTR, 30 min/day at the speed of 11 m/min. The first study (62) using the CatWalK showed a dynamic gait change in EAE mice (including differential front paw and hind paw contact latency), although no exercise effect was observed for the clinical score. The second study (63) used the following protocol regular exercise 32 days prior to EAE induction and 10 days after EAE induction with 6 weeks of dimethyl fumarate (DMF) or glatiramer acetate (GA) treatment. In this study, the mice were assigned to non-exercised (*n* = 45) and exercised (*n* = 46) groups. Motor behavior as a gait parameter was analyzed using the Rotarod. The findings demonstrated that, the day following exercise treatments, the animal groups that underwent exercise and treatment, displayed greater levels of paw pressure, a marker of gait or balance. The GA therapy had no extra impact on dorsal horn microglia/macrophages in rats that had been exercising. Immunoreactivity against astrocytes was diminished globally and significantly in this group. In addition, GA therapy of exercised EAE mice had little impact upon ventral horn glial cells, but enhanced synaptic density cover substantially. The enhanced microglial/macrophage response in the ventral and dorsal horns of the DMF-treated, exercised rats was related to improved clinical symptoms and input density of the synaptic motor neuron (62, 63). It is intriguing that, in the above findings, the effects of exercise seemed to be observed mainly in the gray matter, but not the white matter or adaptive immune system (the main target of EAE); it has been suggested that the gray matter has also been involved in EAE (65). For this purpose, one study employed EAE animals induced by a Cuprizone diet to voluntarily exercise. Voluntary exercise dramatically improved neuromuscular function and motor coordination and elicited early protection against axonal injury and the loss of myelin-associated proteins MBP and 2′,3′-cyclic-nucleotide 3′-phosphodiesterase (CNPase) in the striatum and corpus callosum (66). A surprising level of synaptic plasticity has been documented in the CA1 region of the hippocampus during the acute phase of EAE. Exercise inhibits the loss of parvalbumin-positive (PV +) neurons and restores the expression of normal synaptic plasticity in the CA1 region of the hippocampus of EAE patients. Exercise significantly reduces microgliosis and inflammation, particularly IL-1, and preserves the PV + population, suggesting that the synaptic benefit of exercise reported in the EAE hippocampus can be attributed to its anti-inflammatory/neuroprotective effect. Exercise-induced survival of PV + interneurons modulate the GABAergic effect on EAE synaptic plasticity. In addition, exercise lowers microgliosis in the CA1 region, the expression of tumor necrosis factor (TNF) in microglia, and, to a lesser degree, the hippocampus level of interleukin 1 beta (IL-1β). Intriguingly, exercise exerts a long-lasting assuaging effect on microgliosis that predates its neuroprotective effect, which likely accounts for the enhanced cognitive performance found in pre-symptomatic and acute phase EAE animals (67).

**TABLE 2:** Sample characteristics for studies and neurological signs.

References	Animal species	Type and duration of exercise intervention	Exercise started prior to EAE induction	Exercise started post to EAE induction	Analysis	Results
Patel and White (53)	Female lewis rat, 8 weeks old	Force treadmill running for 10 days	5 days prior induction	10 days post-induction	Clinical disability	Force treadmill running for 10 days did not showed significant difference in clinical disability.
Klaren et al. (52)	Female SJL mice, 8 weeks old	Voluntary wheel running, 50 days. Force treadmill, 36 days		Post-induction	Clinical disability	Voluntary wheel running/50 days and force treadmill/36 days showed no significant difference in clinical disability.
Benson et al. (40)	C57BL/6 female mice, 12 weeks old	Voluntary wheel running, 8 days	1-week prior induction	1-day post-induction	Clinical disability	Voluntary wheel running led to a significant reduction at the onset of clinical sings.
Bernardes et al. (41)	7 weeks old female mice	Swimming, 6 weeks	5 weeks prior induction	10 days post-induction	Clinical disability	6 weeks of forced swimming improves clinical outcome and reduces the pathological markers of EAE in chronic disorders.
Le Page et al. (49)	8 weeks old female Lewis rats	Severs and moderate forced treadmill running, 7 days	2 days prior induction	5 days post-induction	Clinical disability	Mice that were subjected to 2 days of intense treadmill running after disease induction showed a delay in the onset of disease and the day of maximum clinical impairment severity.
Mifflin et al. (45)	Male and female C57BL/6J mice, 6–8 weeks old	voluntary wheel running, 4 weeks		4 weeks post-induction	Clinical disability	The onset of EAE was delayed by roughly 3 days in female mice and approximately 1 day in male mice due to voluntary wheel running. No substantial change in demyelination.
Pryor et al. (46)	C57BL/6J male mice, 10 weeks old	voluntary exercise, 7 days		1-day post-induction	Clinical disability	Exercise modulates disease onset and severity in the EAE, delayed clinical sing, lower disability scores in exercise animal.
Rossi et al. (50)	C57BL/6 female mice, 8 weeks old	Voluntary wheel running, 50 days		From the day of the immunization to 50 days post-induction	Clinical disability	Reduced clinical impairment and synaptic abnormalities in mice with regular exercise.
Souza et al. (47)	C57BL/6 female mice, 6–12 weeks old	Strength training by climbing a ladder, 4 weeks. Endurance training by treadmill, 4 weeks		35 days post-induction	Clinical disability	Protocols for strength and endurance training consistently averted clinical manifestations of EAE and reduced oxidative stress.
Xie et al. (51)	C57BL/6 female mice, 6–8 weeks old	Swimming, 10 weeks	6 weeks prior induction	4 weeks post-induction	Clinical disability	Swimming at a high intensity lowered EAE clinical scores, as well as invading cells and demyelination of spinal cords.
Le Page et al. (48)	Male and female Lewis rat, 8 weeks old	Force treadmill running, 10 days		1-day post-induction	Clinical disability	Exercised mice have a delayed development of clinical impairment and a shorter duration of disease.
Rizzo et al. (67)	C57BL/6 female mice, 5–6 weeks old	Voluntary wheel running, 2 weeks	2 weeks prior induction		Clinical disability	
Shahidi et al. (96)	C57BL/6 female, mice, 6–8 weeks old	Forced swimming, voluntary wheel running, 2 weeks	4 weeks prior induction	4 weeks post-induction	Clinical disability	Regardless of whether the exercise was performed before or after EAE induction, both kinds of exercise (forced Swimming and voluntary wheel running) reduced the intensity of MS symptoms.

## Mechanisms of EAE suppression by exercise

### Regeneration, immune system, demyelination, and exercise in EAE

In MS, immune-mediated demyelination and neurodegenerative alterations unrelated to demyelination have been proposed to contribute to disability (68–70). To assess how exercise training affects the immune system, we identified six studies (Table 3). The study of Rossi et al. (50) was the first study conducting the analysis of potential therapeutic nature of exercises on immune function. Compared to the cannabinoid receptor (CB1) agonist HU210, animals in the VWR group reduced synaptic abnormalities in striatal GABA-mediated spontaneous inhibitory postsynaptic currents. Additionally, exercise completely reversed EAE’s reduction of HU210 responses and prevented EAE-induced loss of dendritic spines in striatal neurons (50). On the day of EAE induction, additional workouts commenced. The research demonstrated that exercise restored synaptic defects in mice, and the authors asserted that exercise had a direct neuroprotective impact.

**TABLE 3:** Sample characteristics for studies for the association between exercise and the immune system, demyelination, regeneration in EAE model.

References	Animal species	Type and duration of exercise intervention	Exercise starts prior of EAE induction	Exercise start post of EAE induction	Results
Rossi et al. (50)	C57BL/6 female 6 mice, 8 weeks old	Voluntary wheel running, 50 days.		From the day of the immunization to 50 days post-induction.	Attenuated dendritic and synaptic defects in exercised.
Bernardes et al. (41)	7-weeks-old female mice	Swimming exercise, 6 weeks	5 weeks prior induction	10 days post-induction	Significant reduction in the number of B cells, CD4 + T cell and CD8 + T cells infiltration into the spinal cord, significant reduction in myelin damage.
Pryor et al. (46)	C57BL/6J female mice, 10 weeks old	Voluntary exercise, 24 h/day, 7 days/week.		1-day post-induction.	Immune cell infiltration and demyelination in the ventral white matter tracts of the lumbar spinal cord were significantly reduced in the EAE exercise group compared with sedentary EAE animals.
Souza et al. (47)	C57BL/6 female mice, 6–12 weeks old	Strength training by climbing a ladder, 4 weeks; endurance training by treadmill, 4 weeks.		35 days post-induction	Strength and endurance training protocols inhibited the production of proinflammatory cytokines interferon (IFN)-γ, interleukin (IL)-17 and IL-1β in the spinal cord of mice with EAE.
Rizzo et al. (67)	C57BL/6 female mice, 5–6 weeks old	Voluntary wheel running, 2 weeks	2 weeks prior induction		Exercise exerts a long-lasting assuaging influence on microgliosis that precedes its neuroprotective action, probably underlying the improved cognitive function observed in pre-symptomatic and acute phase EAE mice.
Einstein et al. (98)	SJL/JCrHsd female mice, 6–7 weeks-old	Force treadmill exercise, 6 weeks.	5 weeks prior induction	1-week post-induction.	Exercise tanning attenuate EAE by modulating the systemic immune system. A total of 2-Exercise increase immune responses to non-specific stimulus. Dose not protects the CNS from encephalitogenic T cells.
Xie et al. (51)	C57BL/6 female mice, 6–8 weeks old	Swimming, 10 weeks.	6 weeks prior induction	4 weeks post-induction	HE training lead to a decrease of IFN-γ and IL-17 and an increase of IL-10 and TGF-β. Analysis of CD4 + T cell subsets from CNS of EAE showed the reduction of Th1 and Th17 populations and an increase of Treg in HE, not ME mice

The second trial indicated that 6 weeks of forced swimming exercise administered prior to the formation of an EAE clinical score may slow the advancement of axonal damage (41), compared with unexercised EAE mice; exercise reduced lymphocyte infiltration, demyelination and axonal damage while decreasing the number of B cells, CD4 + T cells, and CD8 + T cells into the spinal cord. The third study by Pryor et al. (46) showed that VWR for 1 week post-induction reduced autoimmune cell infiltration in the CNS and demyelination in the ventral white matter of the lumbar spinal cord in the EAE-exercise group in comparison to the EAE-sedentary group. In the EAE-sedentary group, neurofilament immunolabeling in the ventral pyramidal and extrapyramidal motor pathways revealed a more random distribution of axons and an apparent loss of axons with a smaller diameter, as well as a larger loss of neurofilaments. In regions of lamina gray matter, laminae I-IX are arranged from dorsal to ventral, whereas lamina X is found centrally around the central canal of the lumbar spinal cord. The sedentary EAE group had a larger loss of motor neurons than the exercising EAE group. These data demonstrate that voluntary exercise reduces and attenuates impairment, decreases immune cell infiltration, and preserves axons and motor neurons in the lumbar spinal cord of EAE animals (46).

Following EAE induction, Souza et al. observed an increase in the pro-inflammatory cytokines interferon (IFN)-[T helper (Th) 1 cytokine], interleukin (IL)-17 (Th17 cytokine), and interleukin (IL)-1 in the CNS, which was reversed by physical activity. A total of 14 days following EAE induction, the proportion of CD4 + CD25 + regulatory T (Treg) cells rose. These findings imply that exercise training may limit the evolution of EAE by reducing pro-inflammatory immune response or by raising the number of Treg cells in peripheral lymphoid tissue (47). In comparison to mice without training, Einstein et al. found that transferring PLP-specific T cells from the lymph nodes of trained mice (6-week treadmill running, 5 days per week, 1 session per day at 23 cm/s) reduced immune cell infiltrations, which in turn resulted in less demyelination and axonal pathology. The authors proposed that exercise training reduces the lymph nodes’ ability to produce effector T lymphocytes after being exposed to the PLP peptide (71).

Low-grade neuroinflammation, including a baseline rise in proinflammatory cytokines and the expression of inflammatory markers on microglia, is linked to aging (72). Infected older animals have less neuroinflammation following exercise, but it was elusive if exercise affects the baseline activation of microglia. A recent study showed examined changes in baseline microglia activation in cells separated from the hippocampus, after exercise using running-wheel (73). Mice who were older had more CD86 and MHC II positive microglia. Aged male mice in the running group showed a decrease in the proportion of CD86 + microglia in the brain, while the aged female mice showed reduced hippocampal CD86 + and MHC II + microglia following access to running-wheel (73). Thus, the results showed that running-wheel access alters microglia activity, but the effects differ depending on the individual factors like the examined brain area, sex, and age.

Yu Xie et al. demonstrated that regular exercise utilizing moderate-intensity swimming training (MEST) had no influence on the overall number of infiltrating cells, Th17 or Treg percentages in the CNS and lymph nodes of EAE mice. On the other hand, high-intensity swimming training (HIST) decreased the clinical manifestations of EAE, and histopathological inhibited pro-inflammatory Th1 and Th17 cells in the CNS of EAE mice while increasing anti-inflammatory Treg cells. In addition, reduction of MOG_35–55_ specific Th1 and Th17 cells, as well as an increase in Treg, were detected in peripheral lymphoid organs as a result of HISE training (51).

### Neurotrophic factors and exercise in EAE

Neurotrophins (NTs) are a large family of dimeric polypeptides, including nerve growth factor (NGF), BDNF, neurotrophin-3 (NT-3), NT-4, NT-5 (74), NT-6, and glial-derived neurotrophic factor (GDNF) (75), that promote growth and the differentiation of neuron development in CNS and PNS as well as the survival of neuronal cells in response to stress (74). They are essential regulators of brain survival, growth, function, and plasticity. As the core tenet of the neurotrophic factor hypothesis, it was proposed that innervation targets release a limited amount of survival factors that serve to maintain a balance between the growth of a target organ and the number of innervating neurons (76). In point of fact, neurotrophic factors have an effect on neuronal activity by fostering the development and maturation of neurons throughout the embryonic stage of life, supporting their health during adulthood, and regenerating neurons following damage (77).

In neurological diseases such as MS, since the dysfunction of synaptic plasticity has been observed in neurological diseases such as MS. There is evidence that NTs have roles in the protection of neurons, the regulation of neuroplasticity, and the preservation of neuronal health and synaptic dysfunction during disease (76). Exercise training influences levels of NTs and, for example, facilitates DNA demethylation in the BDNF gene’s promoter region, raising the production of the signaling protein that activates neurogenesis (78).

Eight studies were identified that studied the effects of exercise training on NT secretion in EAE with mixed outcomes: brain (five studies), spinal cord (one study), serum (two studies) and muscle (one study). Four studies observed significant improvements in neurotropic status (42, 44, 79, 80) while the remaining four reported no significant improvement (52, 53, 81, 82).

In reviewing the exercise effect on EAE, contradictory evidence was observed considering both NTs and neurological outcomes. The most beneficial protocol included daily treadmill running or swimming for at least 14 days prior to induction of EAE, at an intensity of at least 60% maximum workload or 55% of VO_2_ max, for 30 to 60 min each session/day. VO_2_ max, also known as maximal oxygen uptake, is a measurement that determines the greatest amount of oxygen that an individual is capable of utilizing when engaging in physical activity (83). Physical activities have been shown to effectively stimulate hippocampal BDNF, that acts to regulate state of mood states, and improve cognitive functions in MS (84, 85). In contrast, there are studies on effects of exercise on EAE models where neither clinical disability scores nor hippocampus BDNF levels were significantly affected by exercise (52, 53, 81).

## Recommendation, limitation and future directions of exercise on animal models of MS

### Recommendations

Regarding exercise training and EAE, there are undoubtedly numerous avenues for future study. We present a framework for future study in the zone of MS and exercise. Based on the literature knowledge of the different models of EAE that were mentioned previously, in the current review, our recommendations include the following. First, we suggest employing the MOG_35–55_ peptide-induced EAE paradigm in C57BL/6 mice. Because there are specific knockout and transgenic mice available, researchers may examine immune regulation and the pathophysiological effects of inflammation on axonal integrity, C57BL/6 mice have become the most used mouse strain (86). In addition, we advocate the active production of EAE in SJL mice, as this induces an RR illness course that resembles the most prevalent form of MS in people, RR-MS. Because the majority of research on exercise in EAE has been undertaken using monophasic EAE produced with MOG_35–55_ peptide in C57BL/6 mice, it is necessary to employ the RR-EAE model to compare the effects of exercise across animal models. Secondly, the active induction of EAE would be a straightforward starting point that would provide conditions for researchers to study the induction phase of the immune response of EAE concerning exercise training. Following the use of the active EAE model, passive induction will be valuable to study the effector phase of EAE, which will allow investigation of the role of myelin-specific T-cells from donor mice as well as the role of CNS resident cells of the recipient mice (87). Thirdly, we advise investigating various workout dosages and durations. For instance, it may be worthwhile to explore whether frequency (i.e., days of access to a running wheel and a treadmill), intensity (speed and incline of the treadmill), duration, and the time points of exercise commencement are key components of exercise’s disease-modifying effects. Moreover, exercise training protocols using electrophysiology (88, 89) for assessment of results or other treatments is a new developing research focus. Therefore, we recommended using electrophysiology or drug administration in further experiments investigating the role of exercise and MS.

Farrell et al. recently reported that MS patients have a significant incidence of asymmetry in their upper and lower extremities (90). They discovered a relationship between dorsiflexion asymmetry and quality of life, and shoulder flexion asymmetry and lower extremity function. A correlation between plantar flexion and knee flexion asymmetry was also reported with lower extremity functional asymmetry (90). Increased strength of both upper and lower limbs could be a benefit of strength training in MS (91). White et al. (92) revealed the beneficial effects of resistance training on leg strength, mobility, and self-reported weariness and impairment. The knee extensor and plantar flexor muscle forces and walking performance improved significantly (92). The effect of strength training on the physiological variables of animals in the EAE model is limited in the literature. Further research is needed to identify the importance of strength development characteristics in the animal model. These important and unexplained findings can then be transferred to the human model. Working in this field would help researchers interpret adaptations related to unclear involved mechanisms.

### Limitations

Given the complexity of MS, no one animal model can adequately represent the whole range of MS variability in humans or the variety of clinical presentations. To some extent, the pathogenic processes of MS have, however, recently been studied using animal models (93). Despite significant limitations that have an impact on the results and therapeutic application of the literature, the existing research on exercise training and physical activity in EAE is encouraging. As the distinguishing traits of the illness. As the EAE defining characteristics (i.e., inflammatory responses, demyelination, and axon disruption) vary between models, a continual examination of the effects of exercise using multiple EAE models is required (86). Researchers can then evaluate the impact of exercise on specific disease aspects using the best applicable EAE model. Comparison of the impact of exercise on best best-characterized animal models of MS, i.e., the EAE models, TMEV, and toxin-induced demyelination models, will provide a comprehensive understanding of how exercise affects the disease pathogenesis (23). All of these models have already contributed to the present understanding of MS etiology in different ways (23). In particular, EAE is the model which most accurately represents the autoimmune etiology of MS, making it ideal for investigating novel therapies (93). However, since the EAE models only address the immunological components of MS, it may be suggested to use different models of induction to study other aspects of MS (94), for instance, the axonal injury/repair and remyelination process in MS may be better characterized using either the TMEV or toxin-induced demyelination model (23). Another disadvantage of using EAE for exercise intervention is that once animals have clinically been induced with severe EAE, their degree of paralysis limits the capacity to perform physical activity (5). These models could take into account the impacts of exercise and physical activity and be classed as therapeutic interventions at various phases of the disease progression.

## Future directions

### Immunology and neuropathology

More research is needed to unravel the cellular and molecular pathways prompting immunomodulation that underlie the beneficial effects of exercise in MS. Examining immune markers and cytokine modulation will give more evidence regarding the immunological effects of exercise on the CNS and periphery in EAE (70). Examining the effect of exercise on neuropathology using the EAE model is crucial for assessing the efficacy of exercise as a potential adjuvant therapy for MS. Future research should clarify the neuroprotective mechanisms of exercise in MS. Assessing additional neurodegenerative criteria (such as astrocyte or microglia activation and dendritic pathology) will also provide deeper insight into the therapeutic effects of exercise. The mixed outcomes approach on the effects of exercise in modulating the neurobiology of EAE warrants further research.

### Proposing improved exercise protocols in EAE

Another limitation in the published literature is the quantity of exercise used during treadmill conditioning. Variability in treatment stimuli may obscure the actual effects of exercise on mice with severe clinical impairment. The total amount of exercise performed on the running wheel and treadmill is significant for evaluation purposes. The exercise volume may have been insufficient to find differences between the exercise conditions and the inactive condition. Since the optimal intensity of exercise is uncertain, it is suggested that future research quantify exercise intensity by measuring lactate concentrations and/or oxygen uptake (VO_2_) kinetics. Furthermore, a progressive or regressive exercise protocol may be required as clinical manifestations change across the spectrum of EAE progression. It may be necessary to consider altering the intervention to allow animals to move from a forced exercise mode.

### Neurotropic factors

Neurotrophins serve as crucial regulators of neuronal survival, development, function, and plasticity (30). There is a need for additional information regarding the effects of exercise on brain neurotropic factors, such as NGF, GDNF, and BDNF, and their receptors. These variables may play an important role in lesion etiology, neuronal development, and survival (95). Additional research concentrating on the long-term benefits of physical exercise on chronic EAE and the influence of resistance training on muscle contractile characteristics and neurotropic factors, such as BDNF in muscle, would also enhance our knowledge of the therapeutic effects of exercise. Future research should specifically focus on the transit of BDNF and NGF from muscle to the CNS of EAE rats in order to evaluate the genuine neuroprotective properties of the muscle-derived proteins. Before the development of hind limb paralysis, we advise obtaining muscle samples immediately following the training period. Nervous tissue analysis is recommended to study the peripheral nerves and motor end-plates concerning exercise and EAE progression.

## Conclusion

Exercise training results in a beneficial outcome in the physical manifestations of the EAE model. This review analyzed existing reports on the impacts of physical activity and exercise training using EAE models. An insufficient amount of data regarding resistant exercise was also observed. While continued research in this area is required, preliminary findings using EAE models support regular exercise interventions in delaying the progression of the signs and symptoms of neurodegeneration in MS.

## Author contributions

AP, MH, IT, DP, JB, and HZ participated in the conception and design of the study. AS and AP participated in acquiring the data. SB, PS, SD, EŁ, ASt, ŁO, HZ, MA, and IL were responsible for data analysis and interpretation. AP, MH, IT, DP, JB, SB, PS, SD, EŁ, ASt, ŁO, HZ, MA, IL, and AS were responsible for writing and finalization of the manuscript. All authors contributed to the manuscript, approved the submitted version, read, and agreed to the published version of the manuscript.
